# Supplementary material for: Tissue-Level Effect of Andrographis and Ashwagandha Metabolites on Metabolic and Inflammatory Gene Expression in Skeletal Muscle and Adipose Tissue: An Ex Vivo/In Vitro Investigation
Source: Nutrients. 2024 Jul 17;16(14):2291. doi: 10.3390/nu16142291 (PMC11279956; doi:10.3390/nu16142291)
Supplement: Supplementary file 1 [file nutrients-16-02291-s001.zip › nutrients-3068835-supplementary.pdf]

Supplementary material

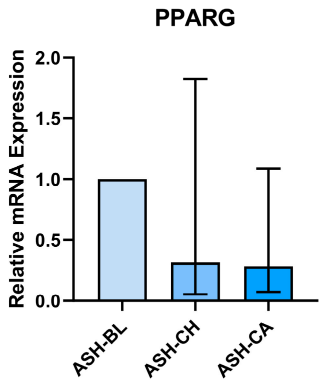

Supplementary Figure S1. Adipose tissue explant *PPARG* mRNA expression is unaltered by treatment with sera of people who supplemented with ashwagandha (ASH). Sera were obtained following 28 days supplementation (chronic, (CH)) and following 28 days supplementation and an acute dose (+1 h) of ASH (chronic + acute (CA)). Values are expressed relative to mRNA expression in samples treated with pre-supplementation baseline (BL) sera. Sera were obtained from 10 participants and pooled for adipose tissue treatment.
